# A Simple Strategy to Reduce Contrast Media Use and Risk of Contrast-Induced Renal Injury during PCI: Introduction of an “Optimal Contrast Volume Protocol” to Daily Clinical Practice

**DOI:** 10.3390/jcdd10090402

**Published:** 2023-09-19

**Authors:** Aiste Zebrauskaite, Greta Ziubryte, Lukas Mackus, Austeja Lieponyte, Evelina Kairyte, Ramunas Unikas, Gediminas Jarusevicius

**Affiliations:** 1 Clinic of Cardiology, Hospital of Lithuanian University of Health Sciences Kaunas Clinics, 50161 Kaunas, Lithuania; greta.ziubryte@kaunoklinikos.lt (G.Z.); lukas.mackus@gmail.com (L.M.); austeja.lieponyte@gmail.com (A.L.); ekairyte2@gmail.com (E.K.); ramunas.unikas@kaunoklinikos.lt (R.U.); gediminas.jarusevicius@kaunoklinikos.lt (G.J.); 2 Faculty of Medicine, Lithuanian University of Health Sciences, 50161 Kaunas, Lithuania; 3 Institute of Cardiology, Lithuanian University of Health Sciences, 50161 Kaunas, Lithuania

**Keywords:** contrast-induced nephropathy, contrast, coronary artery disease, complications

## Abstract

Contrast-induced acute kidney injury is the leading cause of iatrogenic acute nephropathy. Development of contrast-induced nephropathy (CIN) increases the risk of adverse long- and short-term patients outcomes, the hospital costs, and length of hospitalization. There are a couple of methods described for CIN prevention (statin prescription, prehydration, contrast media (CM) clearance from the blood system, and decrease amounts of contrast volume). The CM volume to patient’s creatinine clearance ratio is the main factor to predict the risk of CIN development. The safe CM to creatinine clearance ratio limits have been established. The usage of CM amount depends on personal operators habits and inside center regulations. There is no standardized contrast usage protocol worldwide. The aim of this study was to establish an easy to use, cheap, and efficient protocol to estimate a personalized safe CM dose limit for every patient based on their kidney function. These limits are announced during the “Time Out” before the procedure. Our study included 519 patients undergoing interventional coronary procedures: 207 patients into the “Optimal Contrast Volume” arm and 312 into the control group. Applying the protocol into a daily clinical practice leads to a significant reduction in CM volume used for all type of procedures and the development of CIN in comparison with a control group.

## 1. Introduction

Contrast-induced nephropathy (CIN) is defined as the development of acute kidney injury (AKI) following the administration of contrast media (CM) in the absence of an alternative etiology [1]. Approximately 7% of patients undergoing percutaneous coronary intervention (PCI) experience CIN [2]. In patients with advanced kidney disease, the incidence of CIN increases to around 30% [2,3]. CIN has been strongly linked to adverse clinical outcomes, including death, need for hemodialysis, and increased hospital costs and length of stay [2,3,4,5,6,7]. The ratio of the CM volume used to the creatinine clearance is one of the strongest predictors of the development of CIN following PCI [8,9]. Therefore, in order to reduce the incidence of CIN, it is important to focus on strategies to reduce the volume of CM administered particularly in those with chronic kidney disease (CKD).

CIN remains to be a relatively common complication following PCI, and fear of this complication leads to the underutilization of PCI in high-risk coronary patients with CKD [10,11,12]. Checklists with a time-out stage are widely adapted in cardiac catheterization laboratories and often incorporate the measure of patient’s renal function. However, the use of CM varies greatly among individual operators and cardiac catheterization laboratories. To date, no studies assessing whether incorporating a maximum target for CM that should be not exceeded during PCI into the time-out and monitoring it during the procedure would help to reduce the volume of CM used have not been carried out. To our knowledge, our study is the first to assess this.

The aim of the study was to assess whether the addition of a target for maximum CM volume for the procedure tailored to the patient’s renal function would reduce the contrast volume use during interventional coronary procedures and decrease the risk of CIN development.

## 2. Materials and Methods

We conducted a single-center prospective observational study using the historical control group and the treatment group. The study population included 660 patients who underwent a diagnostic angiogram or elective PCI or a diagnostic angiogram followed by ad hoc PCI at the Clinic of Cardiology, Hospital of Lithuanian University of Health Sciences Kauno Klinikos, between September 2021 and September 2022. Patients with end-stage renal disease undergoing dialysis, patients with New York Heart Failure Association (NYHA) functional class greater than II heart failure, and patients younger than 18 years were not eligible. A total of 141 (21.4%) patients were excluded from the study due to a lack of repeated serum creatinine measurements within 7 days after the interventional procedure.

The “Optimal Contrast Volume Protocol” was developed and introduced in our catheterization laboratory. It includes patient’s renal function described as a serum creatinine level in μmol/L and calculated creatinine clearance (CrCl). Based on the most relevant clinical data [8,9], two target contrast volumes were calculated individually for every patient before the procedure: the maximal CM volume recommended to use and the CM volume that strictly should not be exceeded. The study by Laskey et al. calculated the CM volume-to-CrCl ratio (V/CrCl) for patients undergoing PCI. The CM volume/CrCl ratio of ≥3.7 was a significant and independent predictor of an early abnormal increase in serum creatinine after PCI in an unselected patient population [8]. In the study by Gurm et al., associations between the CM volume and the risk of CIN were assessed. The risk of CIN became significant when the ratio of CM volume-to-CrCl was >2 and was dramatically elevated in patients exceeding a CM volume-to-CrCl ratio of >3. Moreover, it was shown that the CM volume-to-CrCl ratio was superior to MACD in discriminating between patients most likely to develop CIN [9]. In our study, the maximal CM volume recommended to use (in mL) was calculated based on the CrCl by the formula: CrCl × 3.0 [8,9]. The CM volume that strictly should not be exceeded during the procedure (in mL) was calculated using the formula: CrCl × 3.5 [8,9]. If this volume was exceeded, the operator had to consider the discontinuation of the procedure.

**Figure 1:**
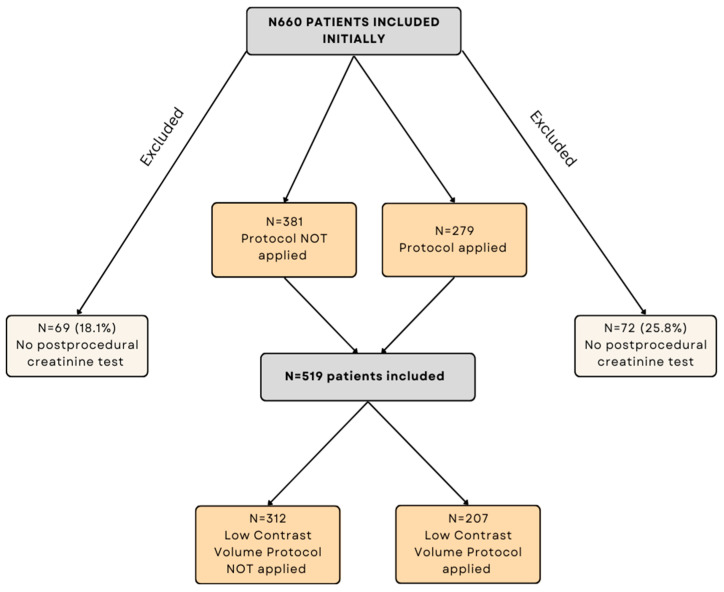
Study flowchart.

Patients who underwent interventional procedures before the introduction of an “Optimal Contrast Volume Protocol” into daily catheterization practice served as a control group (non-protocol group). All the interventional procedures for them were performed without calculating and identifying maximal targeted CM volumes. Patients who underwent interventional procedures after the introduction of the “Optimal Contrast Volume Protocol” into routine catheterization laboratory practice were assigned to the “Optimal Contrast Volume Protocol” group (protocol group). For these patients, the maximal recommended-to-use CM volume and the CM volume that was not recommended to exceed were announced during the time-out phase, and the CM volume was carefully monitored during the procedure. Contrast media “Ultravist 370” (Bayer, Berlin, Germany) was used for all patients. The study flowchart is shown in Figure 1.

The primary endpoint was a reduction of the CM volume using the “Optimal Contrast Volume Protocol”. The secondary endpoint was to assess if the introduction of “Optimal Contrast Volume Protocol” led to a lower risk of CIN development.

Using the serum creatinine level before the procedure as baseline, we calculated CrCl according to the Cockroft–Gault equation [13] and classified patients as having normal baseline renal function (CrCl, ≥60 mL/min), mild CKD (CrCl, 45 to 60 mL/min), moderate CKD (CrCl, 30 to 45 mL/min), or severe CKD (CrCl, <30 mL/min). In the second stage of the analysis, we assessed patients’ serum creatinine levels within 48 h to 7 days after the interventional procedure. If several measurements of serum creatinine levels were collected, the maximal value was assessed. The postprocedural increase in the serum creatinine level was assessed to determine the percentage of patients who developed CIN. CIN was defined following the latest KDIGO criteria: an increase of ≥26.5 μmol/L in the serum creatinine level within 48 h after CM exposure or at least a 1.5-fold increase from baseline within 7 days after CM exposure [14,15]. Patients who had a mild increase in serum creatinine, which did not meet the CIN criteria, were assessed as a separate subgroup.

All the patients are prescribed 500 mL 0.9% isotonic saline before and after the procedure for CIN prophylaxis. For urgent and STEMI patients for whom prehydration before the procedure has not been prescribed, it has to be given during the interventional procedure. Therefore in our study all the patients, historical control group and treatment group, underwent prehydration prophylaxis with isotonic saline.

Data collected included the following: patient’s age, sex, body mass index (BMI), concomitant diseases (arterial hypertension (AH), diabetes mellitus [DM], diagnosed coronary artery disease (CAD), and CKD), serum creatinine level, CrCl, type of interventional procedure performed, CM volume used, and serum creatinine levels after procedure.

The study was conducted in accordance with the Declaration of Helsinki and approved by Kaunas Regional Biomedical Research Ethics Committee (protocol code BE-2-7, dated 22 February 2022).

Normality of data distribution was assessed using the Shapiro–Wilk test. Continuous data were expressed as mean with standard deviation; categorical data, as number and percentage. Comparisons between the groups were performed using the Pearson’s χ^2^ test for categorical variables and the independent Student’s *t* test for continuous variables. Binary logistic regression analysis was applied to identify risk factors for the development of CIN. Variables such as sex (1—male, 2—female), age, baseline serum creatinine, CM volume used, and not applying “Optimal Contrast Volume Protocol” (0—protocol applied, 1—protocol not applied) were included in this analysis. Statistical significance was set at a two-tailed probability level of less than 0.05. Statistical analysis was carried out using SPSS Statistics version 22 (IBM SPSS Inc. Armonk, New York, NY, USA).

## 3. Results

A total of 519 patients were enrolled into the study: 312 patients for whom the “Optimal Contrast Volume Protocol” was not applied and 207 patients for whom the “Optimal Contrast Volume Protocol” was applied during interventional procedures.

The mean age of patients was 69.88 ± 11.74 years; 63.8% of them were male and 36.2% were female. The following comorbidities were documented: AH (*n* = 490, 94.4%), dyslipidemia (*n* = 470, 90.6%), and CAD (*n* = 509, 98.1%). Nearly one-fifth (*n* = 98, 18.9%) of patients had DM and 86 (16.6%) had a history of CKD. The baseline serum creatinine level was 102 ± 16 μmol/L. For 100 (19.3%) patients, a diagnostic angiogram was performed, 61 (11.8%) underwent scheduled PCI, and the majority of patients (*n* = 358, 69.0%) underwent an angiogram followed by ad hoc PCI. The detailed characteristics of patients’ groups and used CM volumes are shown in Table 1. Both the groups were matched for age, sex, BMI, and frequency of comorbidities. There were more men than women enrolled into the study. The proportion of patients with severe CKD was significantly greater in the non-protocol than the protocol group. However, the total number of patients with severe CKD was small; therefore, they were not excluded from the study.

**Figure 2:**
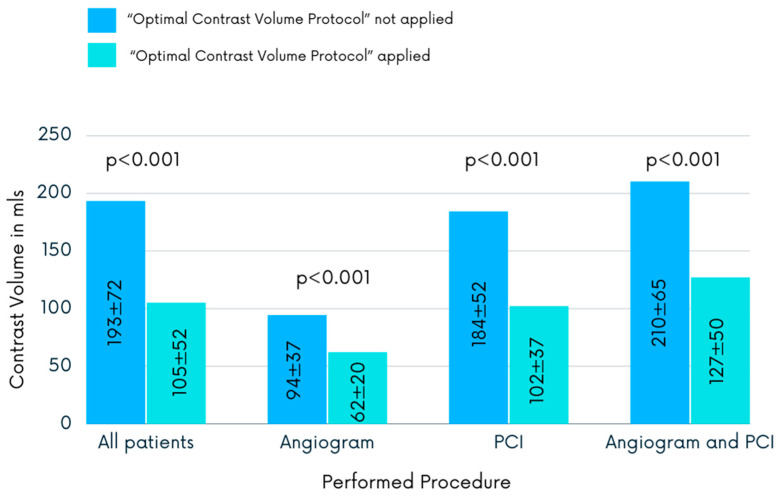
The volumes of contrast media administered during different interventional procedures. The values indicated are means with standard deviation. PCI, percutaneous coronary intervention.

Comparison of the CM volume administered between both groups revealed that in the protocol arm, where the maximal recommended CM volume was calculated and CM use during the procedure was monitored, the volume of used contrast was significantly reduced (192.5 ± 71.9 vs. 104.5 ± 51.7 mL, *p* < 0.001). The same pattern of changes was observed in the angiogram, PCI, and angiogram followed by ad hoc PCI subgroups. Patients who underwent a diagnostic angiogram received the volume of CM reduced nearly by third (93.8 ± 36.6 vs. 61.7 ± 29.0 mL, *p* < 0.001). In the scheduled PCI subgroup, the mean contrast volume was reduced by 45% when the protocol was applied (183.9 ± 52.1 vs. 102.2 ± 36.9 mL, *p* < 0.001). For patients who underwent an angiogram and PCI, the use of the mean contrast volume was reduced by nearly 40.0% (210.0 ± 64.8 vs. 127.2 ± 49.8 mL, *p* < 0.001). Figure 2 shows the volume of the CM administered in both groups via different interventional procedures and its significant reduction in the protocol group.

**Figure 3:**
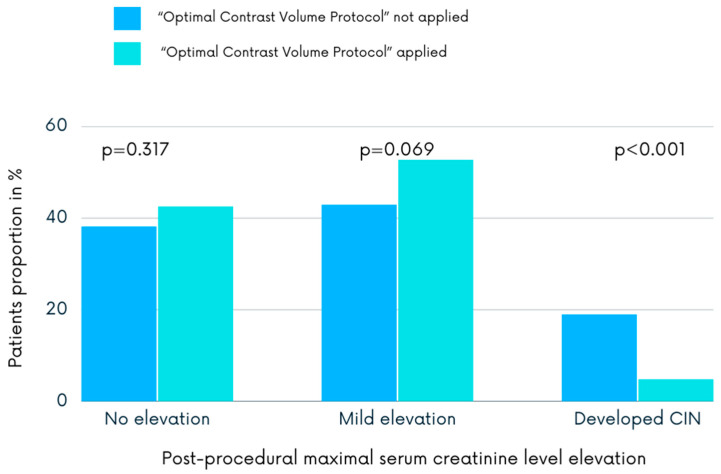
Distribution of patients who had no elevation in the postprocedural serum creatinine levels, who experienced a mild elevation, and who developed contrast-induced nephropathy. CIN, contrast-induced nephropathy.

The proportions of patients who had no increase in the serum creatinine level, who had a mild increase in the serum creatinine level, and who developed CIN after the procedure by group are shown in Figure 3. In the protocol arm, 10 (4.8%) patients developed CIN as compared with 59 (18.9%) in the control group (*p* < 0.001). Of the all study population, 69 (13.5%) patients developed postprocedural CIN, with the majority of them (*n* = 59) being in the non-protocol group (85.5% vs. 14.5%, *p* < 0.001). Patients who developed CIN were more likely not to receive CM according to the “Optimal Contrast Volume Protocol”, to be older, and to have dyslipidemia as well as moderate or severe baseline renal dysfunction (Table 2).

We found that male sex, higher baseline serum creatinine level, and not using the “Optimal Contrast Volume Protocol” were associated with a significantly increased risk of developing CIN. Patients for whom the protocol was not applied were at more than 3-fold greater risk of CIN development (OR = 3.66, 95% CI = 1.66–8.10, *p* = 0.001) (Table 3).

The serum creatinine level did not increase after the procedure in 88 (42.5%) patients of the protocol group compared with 119 (38.1%) patients of the non-protocol (*p* < 0.001). The percentage of patients with a mild serum creatinine elevation, which did not meet CIN criteria, was significantly greater in the protocol than the non-protocol group (*n* = 109, 52.7% vs. *n* = 134, 43.0%, *p* < 0.001).

In total, three patients underwent dialysis; all of them were in the control group. Two of these patients had a severe baseline CKD; one had moderate baseline CKD. For all three patients, the maximal recommended contrast media volume was exceeded.

## 4. Discussion

In this study, we demonstrate that the introduction of “Optimal Contrast Volume Protocol” into routine clinical practice leads to a significant reduction in CM volume administered during interventional cardiac procedures and a significant reduction in CIN rate.

Published studies that assessed the development of AKI after PCI procedures have reported a different incidence of AKI varying from 0.7% to 19% [2,16,17,18,19,20,21,22,23]. This wide variation is considered to be a consequence of several factors. Most of the data published are available from single-center studies. One of the most important discordances among these studies is the usage of different definitions of AKI, and this makes it difficult to compare CIN rates among different studies and populations. The largest multicenter study was conducted by Tsai et al. [2]. More than 985,000 patients who underwent PCI were included in the National Cardiovascular Data Registry Cath-PCI Registry in the US. The study reported that 7.1% of patients developed CIN after PCI was performed. However, this number might have been higher if all patients who underwent PCI had been included into the study. The authors excluded 19.1% of patients without the creatinine level measured before and after PCI and 0.22% of patients with a missing record of CM administered. It can be clearly seen that CIN after interventional procedures is often undiagnosed and underestimated. The results of our study show the same situation as 21.4% of patients were excluded due to a lack of data on a repeated serum creatinine measurement. Furthermore, a considerable number of patients are being discharged on the same or on the next day after the intervention procedure. The majority of these patients do not undergo any creatinine test with their family doctor as recommended. The serum creatinine level peaks in 72 h after CM exposure to the renal system occurs [24]. Due to the pathophysiology of CIN, patients who are discharged on the same day or the next day after the procedure are at risk of underdiagnosed CIN. It is worth noting that there are patients who stay in the hospital longer, but serum creatinine levels are not repeatedly measured despite large volumes of CM used. This shows that better communication among physicians and more escalation of importance of CIN are required.

For a long period of time in the past, there were no universal accredited definition and criteria of AKI and CIN. Different studies and centers used slightly variable criteria. Because of these differences, it was difficult to compare these studies and to summarize findings on CIN. Due to a lack of universal AKI and CIN understanding and varying data on incidence, there was a need to uniform the criteria for AKI. In the last decade, the Acute Kidney Injury Network (AKIN) criteria were formulated to provide a standardized definition and classification of AKI [14,15]. The AKIN criteria have been adopted to daily clinical practice and have been described as the golden standard in the most recent guidelines and recommendations [25,26,27]. These recent AKIN criteria to diagnose CIN were used in our study as well, and this makes the data comparable across other single-center studies.

As CIN is a well-understood process with known short- and long-term consequences, a significance of subclinical creatinine increase remains unclear. Subclinical CIN is defined as an elevation of serum creatinine level, which does not meet the CIN criteria. It can occur in every patient exposed to CM. For a healthy patient, this process may not have any clinical significance because of vigorous tubular repair capability [28]. Under normal circumstances, recovery and regeneration of tubular cells and tubular structure usually takes about 8 to 10 days. However, with a repeated exposure of CM to the kidneys, an abundant destruction of some nephrons that recovery is not possible anymore may occur, and as a final result these nephrons lose their function and are replaced by a fibrotic tissue [29]. Our study showed a significant proportion of patients with a subclinical increase in the serum creatinine level. An increase in the creatinine level varies from a very minimal to a very close to the definition of CIN. We did not detect any importance between the usage of “Optimal Contrast Volume Protocol” and mild postprocedural serum creatinine level elevation. However, a long-term cumulative effect of repeated exposure to CM with subclinical CIN for patients’ renal function remains unclear. This subgroup of patients requires further assessment and long-term follow-up.

Prevention is the key to avoid CIN; however, an early diagnosis and essential treatment can be crucial to achieve better outcomes for these patients. In recent years, a number of significant research studies have been done on assessing early biomarkers of diagnosis and prevention of CIN. Neutrophil gelatinase-associated lipocalin (NGAL) and kidney injury molecule-1 (KIM-1) were found to be early markers of CIN. Data suggest that NGAL and KIM-1 are early predictors of CIN after coronary angiography [30,31]. Antithrombin III might be used as a CIN prediction marker as well [32]. The study by Lacquaniti et al. included 230 patients who underwent bypass surgery. They reported that tissue inhibitor metal proteinase-2, insulin growth factor binding protein-7 (TIMP2*IGFBP7) levels were higher in patients who later developed AKI. The increasement of TIMP2*IGFBP7 was registered in 4 h after surgery; in comparison, the alterations of creatinine and urine output did not occur up to 23 h [33]. Moreover, TIMP2*IGFBP7 levels were found to be associated with the severity of AKI, and certain levels clearly identify patients with further need of renal replacement therapy or contrariwise rule out the need of renal replacement therapy in the next 48 h of observation [33]. The importance of early biomarkers in predicting the development of CIN is substantial in identifying patients at risk. This can reduce the number of cases with undiagnosed AKI and allow identification of patients requiring a specific treatment that could improve outcomes.

Numerous patient-related and procedure-related factors associated with an increased risk of developing CIN have been identified. Patient-related factors include underlying CKD, older age, DM, anemia, and patient’s condition on presentation (cardiogenic shock, acute coronary syndrome, and ST-segment elevation myocardial infarction) [1,22,29,34]. Severe CKD, with an eGFR <30 mL/min/1.73 m^2^, was found to be a risk factor most strongly associated with CIN development, increasing the risk by more than 3 times [2]. The lower baseline kidney function is, the higher is the risk of CIN development [2,8,35]. Due to a high prevalence of CKD among patients with DM, diabetes has also been described as a strong predictor of CIN for a long time. However, the Iohexol Cooperative Study in 1995, a randomized trial involving 1196 patients, showed that DM alone was not independently associated with the risk of developing CIN, but diabetic patients with underlying CKD had a significantly increased risk to develop CIN [36]. The main procedure-related factor is a volume of CM injected to the patient’s circulatory system, and its nephrotoxicity is well known [1,29]. Our data support the fact that the development of CIN is related to the CM volume injected into the bloodstream during the procedure.

There are very few preventive strategies for CIN. Adequate hydration [37,38,39] and minimization of the volume of CM used during the interventional procedures [8,21,40] remain the principal strategies for CIN prevention. The volume of CM injected into the patient’s circulatory system is in proportion to the risk of CIN development [3,8,9,41]. CIN is a multifactorial condition. Patient’s comorbidities, status of hemodynamic insufficiency on presentation, usage of other medications with a possible nephrotoxic effect, and additional patient-specific factors probably interact in different ways for every patient and give a cumulative effect of developing CIN for every patient in a specific manner. However, the findings of the studies mentioned above suggest that the baseline risk of CIN is influenced by the volume of CM. Therefore, the main prevention for CIN development is monitoring and minimizing CM use for every patient specifically. What is more, in order to reduce the risk of CIN development, that complex approach should be widely adopted especially for patients with underlying CKD. This approach includes adequate patient’s pre-procedural preparation, use of strategies minimizing contrast usage, and implementation of modern strategies such as radial access, drug-eluting stents, and finally personalized antithrombotic therapy [42].

Although the need to minimize contrast is generally recognized, for a couple of decades it has remained unclear what CM volume is safe for a patient. Previous studies have suggested the use of maximal acceptable contrast dose (MACD), calculated as 5 mL of CM for every kilogram of body weight (kg) divided by baseline serum creatinine (mg/dL), to describe the threshold of safe CM volume calculated individually for every patient [43]. Despite the fact that MACD was validated by many groups [18,44] it was not widely adopted in daily clinical practice. Furthermore, a significant number of cases of CIN occur even when MACD is not exceeded [39,45]. CM is excreted via kidneys in an unmetabolized state. An alternative method for CM dosing would be calculation of the CM volume based on renal function [8]. Further studies have focused on this method. The study by Laskey et al. included 3179 patients. They calculated the CM volume-to-CrCl ratio for patients undergoing PCI. A CM volume-to-CrCl ratio of ≥3.7 was found to be a significant and independent predictor of an early abnormal increase in the serum creatinine level after PCI in the unselected patient population [8]. Moreover, several years later, the study by Gurm et al. enrolled 58,957 patients undergoing PCI. They assessed the association between the CM volume and the risk of CIN. The risk for CIN approached significance when the ratio of CM volume-to-CrCl exceeded 2 and was dramatically elevated in patients exceeding a CM volume-to-CrCl ratio of 3. Furthermore, it was demonstrated that the CM volume-to-CrCl ratio was superior to MACD in discriminating between patients most likely to develop CIN [9]. Our study design was based on the findings of these two studies.

There is no universal algorithm of the administration of CM during interventional procedures. It varies worldwide and mostly depends on personal operator’s preferences and inside regulations of a clinical center. The aim of introducing the “Optimal Contrast Volume Protocol” to clinical practice is to change operator’s behavior in CM use and encourage all the team to think about every clinical case individually. All team members in a catheterization laboratory are informed about patient’s renal function status, CM volume targets, and restrictions during the time-out. It is a responsibility of every team member to track the volume of the CM administered and communicate it out loud. It makes “Contrast Usage Hygiene” a habit that changes operator’s behavior and improves patient’s safety and outcomes.

Our study describes optimal and upper CM volume limits that are safe for a patient. However, for patients with CKD, the lower CM volume is always the better. The CM volume/CrCl ratio of >2 has been identified as an independent predictor of developing CIN in patients with advanced CKD (eGFR < 30 mL/min/1.73 m^2^) [9]. For patients with CKD and eGFR < 30 mL/min/1.73 m^2^, the CM volume/CrCl ratio of <1 is ideal to minimize the risk of CIN [46,47] and, thus, has been identified as a distinctive characteristic of ultra-low-contrast volume PCI protocols. Therefore, by using the “Optimal Contrast Volume Protocol”, an operator can develop new routine habits of CM use. Furthermore, these skills may be improved further and enable the operator to become a low-contrast and ultra-low-contrast PCI operator aiming to minimize the risk of CIN to minimal.

A time-out procedure before every intervention is daily clinical practice in the majority of catheterization laboratories. The creatinine serum level and CrCl are routinely measured for patients undergoing invasive cardiac procedures; the CM volume-to-CrCl ratio, maximal recommended CV, and CV that should be not exceeded can be calculated effortlessly for every patient. The “Optimal Contrast Volume Protocol” can be easily incorporated into a routine time-out procedure and has the possible implication of impacting patient’s outcomes. Our experience showed that every team member became familiar with it in a short period of time. This simple, cheap, and easy-to-use method contributes to a significant reduction in CM use in all groups of cardiac interventional procedures, resulting in the improved quality of treatment provided and patient’s outcomes.

## 5. Conclusions

The incorporation of “Optimal Contrast Volume Protocol” into daily clinical practice helps statistically significantly reduce the CM volume used during the procedures and significantly decreases the risk of developing CIN.

## 6. Study limitations

This was a single-center study, conducted in the Clinic of Cardiology, Hospital of Lithuanian University of Health Sciences Kauno Klinikos, a center with an active focus on operators’ qualification and improvement in patients’ treatment quality, which might not be representative of a wider population undergoing cardiac interventional procedures in other centers. The study was not blinded and all the operators were aware about the introduction of the “Optimal Contrast Volume Protocol” into daily clinical practice for all patients. The data were collected from the in-hospital system, and the follow-up creatinine levels after discharge from the hospital were collected from the national database that includes all family practices. However, for some patients, serum creatinine levels might have been checked but not entered into the database. Furthermore, it is likely that a number of patients were discharged without measuring their serum creatinine levels and this could lead to underestimation of CIN. The number of patients who were excluded from the study due to a lack of the repeated postprocedural serum creatinine level could contribute to the underestimated incidence of CIN as well. The main purpose of this study was to assess if using an “Optimal contrast volume protocol” reduces CM volume usage for the patients, so we decided to recruit patients with severe CKD as well, because interventional procedures for this group are challenging as CM volume monitoring and decreasing to the minimal limit remains crucial point. We aimed to reflect a real-life cohort and demonstrate that the protocol is suitable to use for patients with different kidney function.

All the patients with heart failure with NYHA functional class >2 were not included into the study due to a higher risk of CIN and different internal protocol for hydration prophylaxis. We have not registered the number of the patients with diagnosis with heart failure in the study database.

## Figures and Tables

**Table 1:** Patients’ characteristics, baseline renal status, and procedure-related characteristics.

Characteristic	Optimal Contrast Volume Protocol		*p*
	Not Applied *n* = 312	Applied *n* = 207	
Demographics			
Age, mean ± SD, years	71.00 ± 12.13	68.20 ± 10.95	0.080
BMI, mean ± SD, kg/m^2^	28.37 ± 4.97	29.14 ± 5.35	0.098
Sex, *n* (%)			
Male	210 (67.3)	121 (58.5)	0.040
Female	102 (32.7)	86 (41.5)	
Comorbidities, *n* (%)			
AH	291 (93.3)	199 (96.1)	0.164
Dyslipidemia	286 (91.7)	184 (88.9)	0.289
CAD	303 (97.1)	206 (99.5)	0.051
DM	57 (18.3)	41 (19.8)	0.661
CKD	58 (18.6)	28 (13.6)	0.306
Baseline renal function			
Baseline serum creatinine, mean ± SD, μmol/L	107.27 ± 76.57	92.27 ± 29.06	0.826
Normal (CrCl ≥ 60 mL/min) *n* (%)	195 (62.5)	143 (69.1)	0.123
Mild CKD (CrCl 46–59 mL/min), *n* (%)	61 (19.6)	38 (18.4)	0.734
Moderate CKD (CrCl 30–45 mL/min), *n* (%)	35 (11.2)	23 (11.1)	0.972
Severe CKD (CrCl < 30 mL/min), *n* (%)	21 (6.7)	3 (1.4)	0.005
Procedure performed, *n* (%)			
Diagnostic angiogram	40 (12.8)	60 (29.0)	<0.001
PCI	31 (10.0)	30 (14.5)	0.112
Diagnostic angiogram and PCI	241 (77.2)	117 (56.5)	<0.001
CM volume, mean ± SD, mL			
Total	192.46 ± 71.88	104.59 ± 51.57	<0.001
Diagnostic angiogram	93.75 ± 36.56	61.67 ± 29.02	<0.001
PCI	183.87 ± 52.13	102.17 ± 36.85	<0.001
Diagnostic angiogram and PCI	209.95 ± 64.79	127.22 ± 49.83	<0.001

BMI, body mass index; AH, arterial hypertension; CAD, coronary artery disease; DM, diabetes mellitus; CKD, chronic kidney disease; CM, contrast media; PCI, percutaneous coronary intervention.

**Table 2:** Comparison of characteristics by the presence and absence of CIN.

Characteristic	CIN after the Procedure		*p*
	No *n* = 450	Yes *n* = 69	
Optimal Contrast Volume Protocol, *n* (%)			<0.001
Not applied	253 (56.2)	59 (85.5)	
Applied	197 (43.8)	10 (14.5)	
Sex, *n* (%)			0.592
Male	285 (63.3)	46 (66.7)	
Female	165 (36.7)	23 (33.3)	
AH	428 (95.1)	62 (89.9)	0.077
Dyslipidemia	412 (91.6)	58 (84.1)	0.047
CAD	440 (97.8)	69 (100)	0.090
DM	89 (19.8)	9 (13.0)	0.183
CKD	81 (18.0)	28 (40.6)	<0.001
Baseline renal function, *n* (%)			
Normal (CrCl ≥ 60 mL/min)	306 (68.0)	32 (46.4)	<0.001
Mild CKD (CrCl 46–59 mL/min)	84 (18.7)	15 (21.7)	0.555
Moderate CKD (CrCl 30–45 mL/min)	44 (9.8)	14 (20.3)	0.010
Severe CKD (CrCl < 30 mL/min)	16 (3.6)	8 (11.6)	0.003
Age, mean ± SD, years	68.99 ± 11.66	75.72 ± 10.58	<0.001
BMI, mean ± SD, kg/m^2^	28.79 ± 5.20	27.88 ± 4.59	0.133
Baseline serum creatinine, mean ± SD, μmol/L	95.35 ± 44.48	117.28 ± 80.79	0.020
Baseline CrCl, mean ± SD, mL/min	78.38 ± 31.89	63.98 ± 30.99	<0.001

CIN, contrast-induced nephropathy; AH, arterial hypertension; CAD, coronary artery disease; DM, diabetes mellitus; CKD, chronic kidney disease; BMI, body mass index; CrCl, creatinine clearance.

**Table 3:** Binary logistic regression analysis of risk factors associated with the development of contrast-induced nephropathy.

Variable	B	SE	Wald	*p*	OR	95% CI
Age	−0.251	0.297	0.715	0.398	0.778	0.434–1.393
Sex	0.049	0.013	14.084	<0.001	1.050	1.023–1.077
Baseline creatinine	0.004	0.002	4.237	0.040	1.004	1.000–1.008
CM volume	0.001	0.002	0.067	0.795	1.001	0.997–1.005
Protocol not applied	1.298	0.405	10.280	0.001	3.663	1.656–8.101
Constant	−6.526	1.058	38.061	<0.001	0.001	

CM, contrast media.

## Data Availability

Research data is stored in Hospital of Lithuanian University of Health Sciences Kaunas Clinics Cardiology Clinic, as per Kaunas Regional Biomedical Research Ethics Committee recommendations.
